# Directed co-evolution of interacting protein–peptide pairs by compartmentalized two-hybrid replication (C2HR)

**DOI:** 10.1093/nar/gkaa933

**Published:** 2020-10-26

**Authors:** Jia Wei Siau, Samuel Nonis, Sharon Chee, Li Quan Koh, Fernando J Ferrer, Christopher J Brown, Farid J Ghadessy

**Affiliations:** p53 Laboratory, Agency for Science, Technology and Research (A*STAR), 8A Biomedical Grove, 138648, Singapore; p53 Laboratory, Agency for Science, Technology and Research (A*STAR), 8A Biomedical Grove, 138648, Singapore; p53 Laboratory, Agency for Science, Technology and Research (A*STAR), 8A Biomedical Grove, 138648, Singapore; p53 Laboratory, Agency for Science, Technology and Research (A*STAR), 8A Biomedical Grove, 138648, Singapore; p53 Laboratory, Agency for Science, Technology and Research (A*STAR), 8A Biomedical Grove, 138648, Singapore; p53 Laboratory, Agency for Science, Technology and Research (A*STAR), 8A Biomedical Grove, 138648, Singapore; p53 Laboratory, Agency for Science, Technology and Research (A*STAR), 8A Biomedical Grove, 138648, Singapore

## Abstract

Directed evolution methodologies benefit from read-outs quantitatively linking genotype to phenotype. We therefore devised a method that couples protein–peptide interactions to the dynamic read-out provided by an engineered DNA polymerase. Fusion of a processivity clamp protein to a thermostable nucleic acid polymerase enables polymerase activity and DNA amplification in otherwise prohibitive high-salt buffers. Here, we recapitulate this phenotype by indirectly coupling the Sso7d processivity clamp to Taq DNA polymerase via respective fusion to a high affinity and thermostable interacting protein–peptide pair. *Escherichia coli* cells co-expressing protein–peptide pairs can directly be used in polymerase chain reactions to determine relative interaction strengths by the measurement of amplicon yields. Conditional polymerase activity is further used to link genotype to phenotype of interacting protein–peptide pairs co-expressed in *E. coli* using the compartmentalized self-replication directed evolution platform. We validate this approach, termed compartmentalized two-hybrid replication, by selecting for high-affinity peptides that bind two model protein partners: SpyCatcher and the large fragment of NanoLuc luciferase. We further demonstrate directed co-evolution by randomizing both protein and peptide components of the SpyCatcher–SpyTag pair and co-selecting for functionally interacting variants.

## INTRODUCTION

Cellular biology is governed by a complex network of protein–protein interactions (PPIs). In many cases, the principal interacting component of one protein in a binary complex presents as a short, often alpha helical region, that retains binding affinity in the form of a discrete peptide (1,2). This knowledge can guide development of both small molecule and peptidic antagonists towards therapeutic targets and protein biosensors (3–6). Protein engineering can further derive novel peptide–protein pairs by splitting compliant proteins into interacting components. This approach has yielded robust tools for biosensing, imaging and targeted protein conjugation (7–9). Methodologies that disclose new PPIs, modulate affinities of known PPIs, and select for novel peptide/protein binders are therefore important tools for proteomics, drug discovery, target validation and biotechnology applications. To this end, a suite of ‘N-hybrid’ platforms including the prototypical yeast two-hybrid (Y2H) selection methodology have been developed and successfully implemented over the years (10–13). These couple *in vivo* protein–protein interactions to co-localization of two protein domains required for signal generation, typically a component of the transcriptional machinery and a DNA-binding protein. Despite the wide-spread success of conventional *in vivo* two-hybrid platforms, certain limitations remain. Efficient nuclear import of the fusion proteins is often a prerequisite for read-out, and reliance on cell viability along with use of mesophilic reporter proteins limits use for co-selection of thermostability, a desired feature in many downstream applications of evolved proteins. The protein-fragment complementation assay (PCA) is a related genetic *in vivo* method, wherein a PPI leads to reconstitution of an otherwise nonfunctional split transducing/reporter protein (14–17). PCAs can provide dynamic read-outs and are often amenable to high-throughput screening campaigns. They can, however, be prone to background issues due to spontaneous reassembly of the split fragments that is independent of fusion partner interaction. As with two-hybrid approaches, most PCAs employ mesophilic reporters, again restricting their use in co-selection of thermostability.

The compartmentalized self-replication (CSR) directed evolution platform was originally developed to select for thermostable nucleic acid polymerase variants with improved functionality (18). CSR entails clonal encapsulation of bacteria expressing a library of polymerase variants into the aqueous compartments of a heat-stable emulsion. Subsequent thermal cycling permits amplification of a polymerase gene only by the particular enzyme it encodes, quantitatively linking activity of constituent library members to the copy number of their respective genes. This dynamic feature enables rapid selection of novel polymerases with desired properties such as improved thermostability, tolerance for non-natural bases, and resistance to inhibitors (19–22). CSR has been further modified to permit selection of other enzymes by coupling their activities to polymerase read-out (18,23). Here, we show that a high affinity and thermostable peptide–protein interaction can also be coupled to DNA polymerase function, thus enabling read-out of their encoding genes by CSR. This is achieved by expression of candidate peptides/proteins as respective fusions to Taq polymerase and the Sso7d processivity clamp. Peptide–protein interaction brings Sso7d into close proximity with Taq polymerase, allowing DNA amplification in otherwise prohibitively high-salt concentrations. This approach, termed compartmentalized two-hybrid replication (C2HR) is used in selections employing well-characterized high-affinity protein–peptide pairs (SpyCatcher-SpyTag and the large/small fragments of split NanoLuc luciferase). C2HR also permits co-evolution of interacting protein–peptide pairs, as exemplified by co-randomization of SpyCatcher and SpyTag and selection for interacting variants.

## MATERIALS AND METHODS

### Materials

Oligonucleotides and genes were from Integrated DNA Technologies; restriction enzymes, T4 polynucleotide kinase and T4 DNA ligase were from NEB; Pfu DNA polymerase (Agilent Technologies) and Taq DNA polymerase (Bioline) were used for DNA amplification. Nucleic acid purification kits were from Qiagen and chemicals from Sigma. Electrocompetent TG1 and BL21 cells were obtained from Lucigen.

### Oligonucleotides

See Table 1.

**Table 1.** Oligonucleotides

Name	Sequence (5′ - 3′)
TAQNde1-F	AAGGAGATATACATATGCGCGGCATGCTGCCACT
TAQXho1R	GGTGGTGGTGCTCGAGTCATTCCTTGGCACTCAGCCAATCTTC
pET-ATG-R	CATATGTATATCTCCTTCTTAAAGTTAAAC
Stoff-F	AGCCCAAAAGCGCTG
HhH-Stoff-F	AAGGAGATATACATATGAAGTCGGGCCGTCAGGAG
HhH-GGG-Stoff-R	GCTTTTGGGCTCATACCTCCGCCTACGTCGTAGGCACCGCGAAGCTTACGTCTGATG
StoffAPWP-F	GCCCCGTGGCCACCTC
KALEtoLPETGGG-R	TTCGCCTCCACCTGTTTCCGGCAGTGGGCTTTCCAAGAGCCCGAAC
SS07DINF-F	GGAGATATACATATGGCAACCGTTAAATTCAAGTATAAGG
SSO7DINF-R	ACCTGTTTCCGGCAGACTACCTTTTTTTTGCTTTTCCAGCA
EXOLPETV2-F	CTGCCGGAAACAGGTGGAG
Nde1-Stoff-F	AAGGAGATATACATATGGGAGGCGAAGCCCCG
Xho1-Stoff-R	TTTCTTTACCAGACTCGAGTCATTCCTTGGCAC
SSO7D-BAM-pDUET-F2	ACCACAGCCAGGATCCaGCAACCGTTAAATTCAAGTATAAGG
SSO7D-Sort-SalI-pDUET-R	CCGCAAGCTTGTCGACTTATCCTCCAGTCTCAGGCAGGCTCCCACCACCACTACCTTTTTTTTGCTTTTCCAGCATTTGCAG
DUETHHH-F	ATAATGCTTAAGTCGAACAGAAAGTAATCGTATTG
DUETHHH-R	TCCTCCAGTCTCAGGCAGTACGT
SPYCDUET-F2	CCTGAGACTGGAGGATCTGTCGAC GATTCAGCCACCCACATTAAATTTAGTAAACGCGA
SPYCHHH-R2	CGACTTAAGCATTATGAATTCTTAGCCGTTAACTGTGACCTGTCCCTGTTCATT
SPYTINF-TOP	AAGGAGATATACATATGGGAGCTCACATCGTGATGGTGGACGCATATAAGCCGACTAAGGGATCTACTAGTTCTATGGGAGGCGAAGC
SPYTINF-B	GCTTCGCCTCCCATAGAACTAGTAGATCCCTTAGTCGGCTTATATGCGTCCACCATCACGATGTGAGCTCCCATATGTATATCTCCTT
NanoBigDUET-F	CCTGAGACTGGAGGATCTGTCGACATGGTCTTCACACTCGAAGATTTCGTTGG
NanoBigHHH-R	CGACTTAAGCATTATGAATTCTTAACTGTTGATGGTTACTCGGAACAGCATG
NanoSmallV2-TOP	AAGGAGATATACATATGGTAACCGGTTACCGTTTGTTCGAAGAGATTTTGGGATCTACTAGTTCTATGGGAGGCGAAGC
NanoSmallV2-BTM	GCTTCGCCTCCCATAGAACTAGTAGATCCCAAAATCTCTTCGAACAAACGGTAACCGGTTACCATATGTATATCTCCTT
NanoStrongV2-TOP	AAGGAGATATACATATGGTATCAGGTTGGCGTTTGTTCAAGAAGATTAGCGGATCTACTAGTTCTATGGGAGGCGAAGC
NanoStrongV2-BTM	GCTTCGCCTCCCATAGAACTAGTAGATCCGCTAATCTTCTTGAACAAACGCCAACCTGATACCATATGTATATCTCCTT
NanoStrongAV2-TOP	AAGGAGATATACATATGGTAACCGGTTACCGTTTGTTCGAAAAGATTAGCGGATCTACTAGTTCTATGGGAGGCGAAGC
NanoStrongAV2-BTM	GCTTCGCCTCCCATAGAACTAGTAGATCCGCTAATCTTTTCGAACAAACGGTAACCGGTTACCATATGTATATCTCCTT
NanoStrongBV2-TOP	AAGGAGATATACATATGAACGTAACCGGTTACCGTTTGTTCAAGAAGATTAGCAACGGATCTACTAGTTCTATGGGAGGCGAAGC
NanoStrongBV2-BTM	GCTTCGCCTCCCATAGAACTAGTAGATCCGTTGCTAATCTTCTTGAACAAACGGTAACCGGTTACGTTCATATGTATATCTCCTT
NanoStrongCV2-TOP	AAGGAGATATACATATGAACGTATCAGGTTGGCGTTTGTTCAAGAAGATTAGCAACGGATCTACTAGTTCTATGGGAGGCGAAGC
NanoStrongCV2-BTM	GCTTCGCCTCCCATAGAACTAGTAGATCCGTTGCTAATCTTCTTGAACAAACGCCAACCTGATACGTTCATATGTATATCTCCTT
NanoStrongDV2-TOP	AAGGAGATATACATATGGGTGTAACCGGTTGGCGTTTGTGCGAACGTATTTTGGCCGGATCTACTAGTTCTATGGGAGGCGAAGC
NanoStrongDV2-BTM	GCTTCGCCTCCCATAGAACTAGTAGATCCGGCCAAAATACGTTCGCACAAACGCCAACCGGTTACACCCATATGTATATCTCCTT
MStoffV2-TOP	AAGGAGATATACATATGGGAGGATCTACTAGTTCTATGGGAGGCGAAGC
MStoffV2-BTM	GCTTCGCCTCCCATAGAACTAGTAGATCCTCCCATATGTATATCTCCTT
LPETGG-Sal1-F	TGCCTGAGACTGGAGGATCTGTCGAC
SPYTR6.2	CATAGAACTAGTAGATCCCTTAGTCGGCTTATATGCGTCMNNMNNMNNMNNGTGAGCTCCCATATGTATATCTCCTTCTTATACTTAACTAATATAC
SPYTR5.2	CATAGAACTAGTAGATCCCTTAGTCGGMNNMNNMNNGTCCACCATCACGATMNNMNNMNNCATATGTATATCTCCTTCTTATACTTAACTAATATAC
SpyC-NNK1-R	CAGTAGCCACTTCATACCCGTCCGGTGCGGCGGTTTCCACMNNGGTGTATTTACCTGGGTACAGGT
SpyC-NNK2-F	GACGGGTATGAAGTGGCTACTGCAATTACTNNKACCGTAAATGAACAGGGACAGGTCACAG
SpyT-NNK-R	CATAGAACTAGTAGATCCCTTAGTCGGCTTATATGCGTCCACCATCACMNNGTGAGCTCCCATATGTATATCTCCTTCTTATACTTAACTAATATAC
INF-Pet22-JW-SSO-SPYC-F	AAGGAGATATACATATGGGCAGCAGCCATCACCATCATCACC
INF-Pet22-JW-SSO-SPYC-R	GACGGAGCTCGAATTCTTAGCCGTTAACTGTGACCTGTCCCTGTTCATTTAC
INF-Pet22-JW-SPYT-F	AAGGAGATATACATATGGGAGCTCACATCGTGATGGTGGACGC
INF-Pet22-JW-SPYT-R	GGTGGTGGTGCTCGAGTTCCTTGGCACTCAGCCAATCTTCGCC
BIOOLS79-duetMCS2-F	BIOTIN-GTAAGCTGGAAGTTGTTGCTGCGTGAGCGGATAACAATTCCCCATCTTAG
SpyTag-SpeI-R2	TTCGCCTCCCATAGAACTAGTAGATCC
BIO-OLS79-LPETGG-Sal1-F	BIOTIN-GTAAGCTGGAAGTTGTTGCTGCTGCCTGAGACTGGAGGATCTGTCGAC
NESTSpyTag-SpeI-R3	GCCTCCCATAGAACTAGTAGATCCCTTAGTC
NESTOLS79-duetMCS2-F	GAAGTTGTTGCTGCGTGAGCGGAT
NESTOLS79-LPETGG-F	GAAGTTGTTGCTGCTGCCTGAGAC
NESTSpyTag-SpeI-R4	TCCCATAGAACTAGTAGATCCCTTAGTCGG
spyT-F19	CAAGCAGAAGACGGCATACGAGATGCCAATGTGACTGGAGTTCAGACGTGTGCTCTTCCGATCTAGCTGTATATTAGTTAAGTATAAGAAGGAGATATACATATG
spyT-R19	AATGATACGGCGACCACCGAGATCTACACTCTTTCCCTACACGACGCTCTTCCGATCTAGCTCATAGAACTAGTAGATCCCTTAGTCGG
spyT-F17	CAAGCAGAAGACGGCATACGAGATGCCAATGTGACTGGAGTTCAGACGTGTGCTCTTCCGATCTACGTGATATACATATGGGAGCTCAC
spyT-R17	AATGATACGGCGACCACCGAGATCTACACTCTTTCCCTACACGACGCTCTTCCGATCTACGTAGATCCCTTAGTCGGCTTATATGCGTC

### Vector construction

Taq pET22b(+) was generated via amplification of the Taq polymerase gene with primers TAQNde1-F and TAQXho1R, followed by infusion into pET22b(+) via NdeI and XhoI sites. Inverse PCR was carried out on Taq pET22b(+) with primers pET-ATG-R and Stoff-F, followed by intramolecular ligation to produce Stoffel pET22b(+), which encodes only the Stoffel fragment. HhH-Stoffel pET22b(+), which encodes for Topoisomerase V HhH processivity domain-Stoffel fusion, was produced via amplification of the processivity domain gene using primers HhH-Stoff-F and HhH-GGG-Stoff-R, followed by infusion into Stoffel pET22b(+) via NdeI site. Inverse PCR and intramolecular ligation were carried out on Taq pET22b(+) with primers StoffAPWP-F and KALEtoLPETGGG-R to generate Exo-Stoffel pET22b(+). Sso7d-Stoffel pET22b(+), encoding for Sso7d-Stoffel fusion, was constructed via infusion cloning Sso7d gene with primers SSO7DINF-F and SSO7DINF-R into an inverse PCR product from amplification of Exo-Stoffel pET22b(+) generated using primers pET-ATG-R and EXOLPETV2-F. Stoffel pETDuet-1 was constructed by subcloning stoffel fragment from Stoffel pET22b(+) with primers Nde1-Stoff-F and Xho1-Stoff-R into the second multiple cloning site (MCS) of pETDuet-1 via NdeI and XhoI sites. Sso7d was introduced into the first MCS of pETDuet-1 using primers SSO7D-BAM-pETDuet-1 -F2 and SSO7D-Sort-SalI-pETDuet-1-R on Stoffel pET22b(+). This produces Sso7d Stoffel pETDuet-1. Inverse PCR was carried out on Sso7d Stoffel pETDuet-1 with primers DUETHHH-F and DUETHHH-R, followed by infusion of SpyCatcher gene (residues 22–101) using primers SPYCDUET-F2 and SPYCHHH-R2. This gives Sso7d-SpyCatcher Stoffel pETDuet-1. Complementary primer pair SPYTINF-TOP and SPYTINF-B were annealed to form an oligo duplex which was cloned into Sso7d-SpyCatcher Stoffel pETDuet-1 via NdeI site to yield Sso7d-SpyCatcher SpyTag-Stoffel pETDuet-1.

The large fragment of split NanoLuc luciferase was amplified using primers NanoBigDUET-F and NanoBigHHH-R and the product cloned into Sso7d Stoffel pETDuet-1 to create Sso7d-NB Stoffel pETDuet-1. A series of complementary primer pairs were annealed to form oligo duplexes which were cloned into this vector to get Sso7d-NB NS1/2/3/4/5/6-Stoffel pETDuet-1 for test selection.

Lib 1 and Lib 2 were created by amplifying Sso7d-SpyCatcher Stoffel pETDuet-1 with primers LPETGG-Sal1-F and SPYTR6.2, and LPETGG-Sal1-F and SPYTR5.2, respectively. Lib 3 was created by overlap extension PCR of two PCR products—the first with primers LPETGG-Sal1-F and SpyC-NNK1-R and the second with primers SpyC-NNK2-F and SpyT-NNK-R on the same vector. All resultant library PCR products were then cloned into Sso7d-SpyCatcher Stoffel pETDuet-1 via SalI and SpeI.

Constructs for expression and purification of Sso7d-SpyCatcher and SpyTag-Stoffel fusion proteins were created by amplifying Sso7d-SpyCatcher SpyTag-Stoffel pETDuet-1 using primer pairs INF-Pet22-JW-SSO-SPYC-F and INF-Pet22-JW-SSO-SPYC-R, and INF-Pet22-JW-SPYT-F and INF-Pet22-JW-SPYT-R and the subsequent respective PCR products infused into pET22b(+).

### Polymerase activity assays

Constructs expressing Stoffel, HhH-Stoffel fusion protein and Sso7d-Stoffel were transformed into *Escherichia coli* BL21 (DE3) competent cells. Cells expressing HhH-Stoffel were grown in LB medium with glucose (10 mM) and induced for 3 h at 37°C with 1 mM isopropyl-β-D-thiogalactoside (IPTG). Cells expressing Sso7d-Stoffel were grown in LB medium with glucose (10 mM) and induced with 1 mM IPTG with different temperature and duration as described in text. About 1 ml of culture was then harvested by centrifugation, washed with phosphate buffered saline (PBS) twice and resuspended in 50 μl of PBS. About 2 μl of cell suspension was used for PCR (95°C for 5 min, 25 cycle of 95°C for 5 s, 55°C for 30 s, and 72°C for 1 min) with 10 ng pET22b(+) and 0.5 μM of each primer petF2 and pET-ATG-R. PCR reactions involving HhH-Stoffel were carried out with PCR reaction buffer containing 30 mM Tris pH 8.0 and 0.2% Tween 20 while reactions for Sso7d-Stoffel were carried out with PCR reaction buffer comprising of 10 mM Tris-HCl pH 8.3, 10 mM KCl, 1.5 mM MgCl_2_. Differentiation of polymerase activity was carried out by adjustment of salt concentration using KCl. Successful polymerase activity yields a 198 bp amplicon using expression plasmid as template. Subsequently selected SpyTag variants were screened and compared using the same method. Activity assays for PCR of the larger 1545 bp fragment directly using expressor cells was carried out essentially as above using 10 ng pET22-SBPp53delta plasmid template and 0.5 μM of each primer petF2 and petR. Normal cycling conditions were 95°C for 5 min (1 cycle), 95°C for 5 s, 60°C for 30 s, and 72°C for 120 s (25 cycles). Accelerated cycling parameters were 95°C for 5 mins (1 cycle), 95°C for 5 s, 60°C for 15 s, and 72°C for 10 s (35 cycles) Using recombinant proteins, the following cycling parameters were used: 95°C for 5 mins (1 cycle), 95°C for 5 s, 60°C for 15 s, and 72°C for 15 s (accelerated) or 120 s (normal) (35 cycles).

### Compartmentalized self-replication (CSR) selections

CSR reactions were essentially carried out as previously described (18). All expressor cells were grown in LB medium with glucose (10 mM). 200 μl of aqueous phase consisting of Stoffel buffer (10 mM Tris-HCl pH8.3, 10 mM KCl and 1.5 mM MgCl_2_), 0.25 mM dNTPs, 0.5 μM of each primer, 100 mM KCl, 1 mg/ml bovine serum albumin (BSA), 1 × 10^7^*E. coli* BL21 (DE3) expressor cells were manually dispersed (1 drop every 5 s) into 400 μl of oil phase [4.5% (v/v) Span 80, 0.45% (v/v) Tween 80 and 0.05% Triton X-100 (v/v) in mineral oil] with constant stirring at 1250 rpm. Stirring was continued for 9 min before thermocycling. CSR was carried out using different primers pairs (BIOOLS79-duetMCS2-F and SpyTag-SpeI-R2 for test selection, BIO-OLS79-LPETGG-Sal1-F and SpyTag-SpeI-R2 for selection of Lib 1 and 3, BIO-OLS79-LPETGG-Sal1-F and NESTSpyTag-SpeI-R3 for selection of Lib 2) at 95°C for 5 min, followed by 10 cycles of 95°C for 5 s, 55°C for 30 s and 72°C for 1 min. The aqueous phase was extracted twice with 900 μl ether and treated with 10 μl exonuclease and 2 μl Dpn1 overnight at 37°C. The aqueous phase was then incubated with 25 μl streptavidin M280 beads (Invitrogen) for 1 h with rotation at room temperature before three washes with 200 μl of PBSBT [PBS + 0.1% (w/v) BSA, 0.1%(v/v) Tween 20] and three washes with 200 μl of PBS. The beads were then resuspended with PCR reactions containing different primer pairs (NESTOLS79-duetMCS2-F and SpyTag-SpeI-R2 for test selection, NESTOLS79-LPETGG-F and SpyTag-SpeI-R2 for selection of Lib 1 and 3, NESTOLS79-LPETGG-F and NESTSpyTag-SpeI-R4 for selection of Lib 2) and subjected to a rescue PCR (95°C for 5 mins followed by 20 cycles of 95°C for 5 s, 55°C for 20 s and 72°C for 1 min).

### Sequence analysis

Amplicons generated by C2HR were adapted by PCR using primers SpyT-F19 and SpyT-R19 (Lib 1) and SpyT-F17 and SpyT-R17 (Lib 2) and sequencing carried out using the NextSeq Illumina platform (DNA Link, Korea). Data extraction/analysis was carried out using Python scripts developed in the p53 Laboratory.

### Protein expression and purification

The Sso7D-SpyC construct was cloned with a N-terminal 6xHis-tag and transformed into *E. coli* BL21(DE3) (Invitrogen) competent cells. These were grown in LB medium with glucose (10 mM) at 37°C and induced at OD_600 nm_ ∼ 0.6 at 25°C with 1 mM IPTG and incubated overnight. Cells were then harvested by centrifugation, and the cell pellet was resuspended in binding buffer (50 mM Tris pH 8, 500 mM NaCl, 20 mM Imidazole) and sonicated. The cell lysate was heated at 65°C for 15 min before clarification by centrifugation. The clarified cell lysate was applied to a 1 ml His-TrapFF column (GE Healthcare) pre-equilibrated in binding buffer and bound protein was eluted using a linear gradient (0–100%) in elution buffer (50 mM Tris pH 8, 500 mM NaCl, 1 M imidazole) over 50 column volumes. The fractions containing the protein were pooled and dialyzed into buffer A solution (20 mM Tris, pH 8, 1 mM DTT) using HiPrep 26/10 desalting column, and then loaded onto an anion-exchange Resource Q 1 ml column (GE Healthcare) pre-equilibrated in buffer A. The column was then washed in six column volumes of buffer A and bound protein was eluted with a linear gradient in buffer comprising 1 M NaCl, 20 mM Tris pH 8, and 1 mM DTT over 30 column volumes. Protein purity as assessed by SDS-PAGE was ∼95%, and the protein was concentrated using Amicon-Ultra (3 kDa MWCO) concentrator (Millipore).

The SpyTag-Stoffel construct was cloned with a C-terminal 6× His-tag and transformed into *E. coli* BL21(DE3) (Invitrogen) competent cells. These were grown in LB medium at 37°C and induced at OD_600 nm_ ∼ 0.6 at 30°C with 0.5 mM IPTG and incubated overnight. Cells were then harvested by centrifugation, and the cell pellet was resuspended in binding buffer (50 mM Tris pH 8, 500 mM NaCl, 20 mM imidazole) and sonicated. The cell lysate was then heated at 65°C for 15 min and clarified by centrifugation. The clarified cell lysate was applied to a 1 ml His-TrapFF column (GE Healthcare) pre-equilibrated in binding buffer and bound protein was eluted using a gradient elution (0–100%) in elution buffer (50 mM Tris pH 8, 500 mM NaCl, 1 M imidazole) over 50 column volumes. The fractions containing the protein were pooled and buffer exchanged into buffer with 50 mM Tris pH 8, 150 mM NaCl, 1 mM DTT and run on a size exclusion HiLoad 16/600 Superdex S200 column. Fractions were pooled and protein purity as assessed by SDS-PAGE was ∼95%. The protein was concentrated using Amicon-Ultra (10 kDa MWCO) concentrator (Millipore).

Activity assay was carried out by co-incubating purified proteins (Sso7D-SpyC and SpyTag-Stoffel, 5 μM each) at room temperature for 30 min in buffer comprising 50 mM Tris pH 8, 150 mM NaCl. About 1 μl of the reaction mixture was subjected to polymerase activity assays as mentioned above.

### Pull-down assay

Biotin-labelled peptides (100 μM) were incubated with streptavidin beads (50 μl) for 2 h in PBS at room temperatures prior to washing with three washes of PBS + 0.1% (v/v) Tween 20. Beads were next incubated at 4°C overnight with 500 μM of Sso7d-SpyCatcher protein, followed by three washes with PBS + 0.1% (v/v) Tween 20 and then three washes with PBS. Bound protein was eluted by boiling in SDS buffer prior to analysis by SDS-PAGE.

## RESULTS

### Coupled polymerase read-out of protein–peptide interactions using model interactants

**Figure 1.**
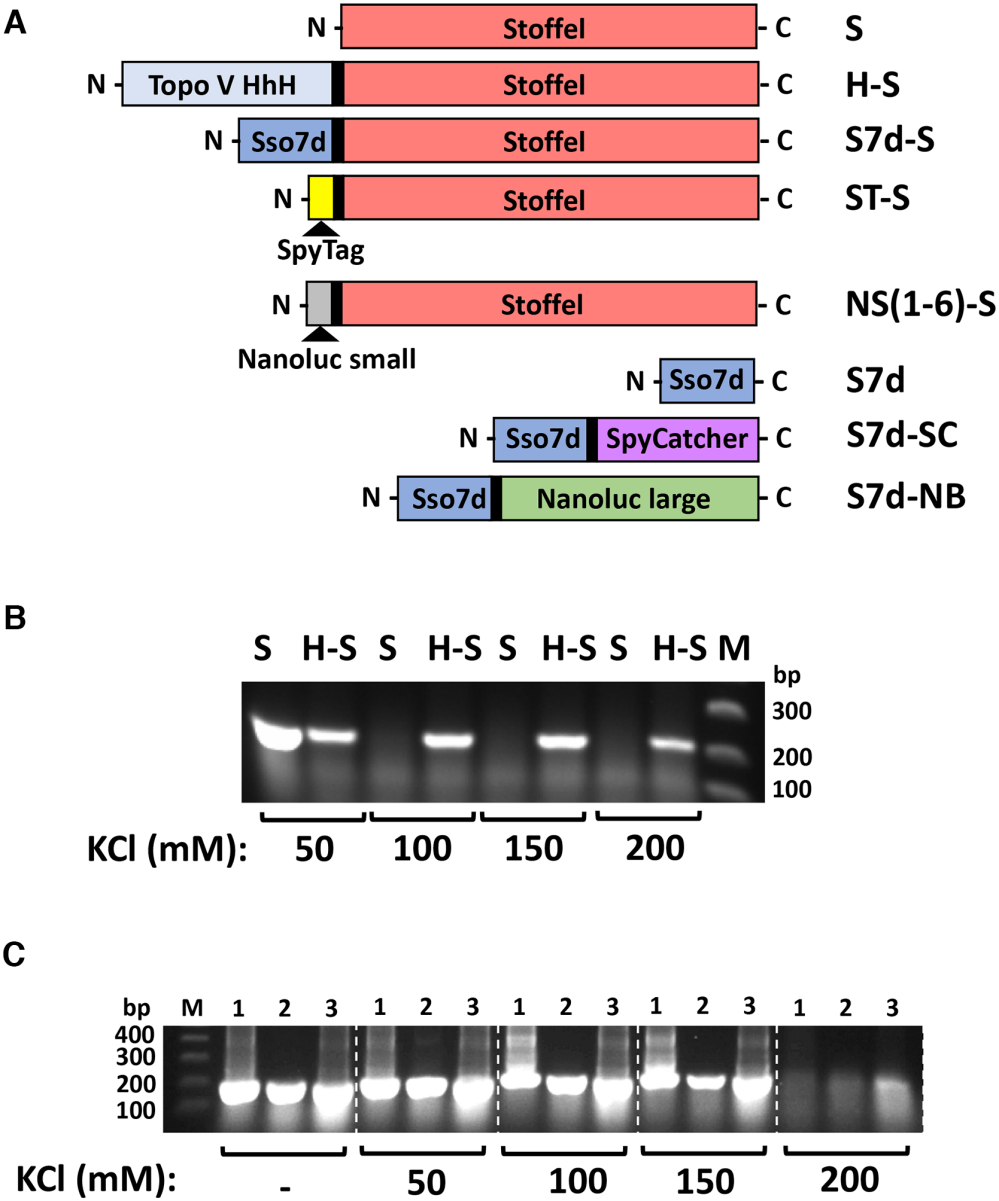
Processivity-clamp fusion enhances polymerase activity in high-salt buffer conditions. (**A**) Schematic of expression constructs and abbreviated names used in this study. (**B**) PCR amplicon yields at indicated KCl concentrations in reaction buffer using *Escherichia coli* cells expressing either Stoffel fragment (S) or a Topoisomerase V HhH processivity domain-Stoffel fusion protein (H-S). (**C**) Same as in (**B**) using *E. coli* cells expressing Sso7d-Stoffel fusion protein with induction at 37°C for 3 h (lane 1), 37°C overnight (lane 2) and room temperature overnight (lane 3); *n*=2 (replicate data shown in Figure 2A). Similar results have been reported previously (24–25).

We first assayed the polymerase activity of the Stoffel fragment of Taq DNA polymerase (amino acids 293–832) (24) fused to either the Sso7d or Topoisomerase V HhH processivity domains. As previously reported (25,26), both domains facilitated PCR amplification in higher salt concentrations (>50 mM KCl) that inhibited the non-chimeric Stoffel fragment (Figure 1).

**Figure 2.**
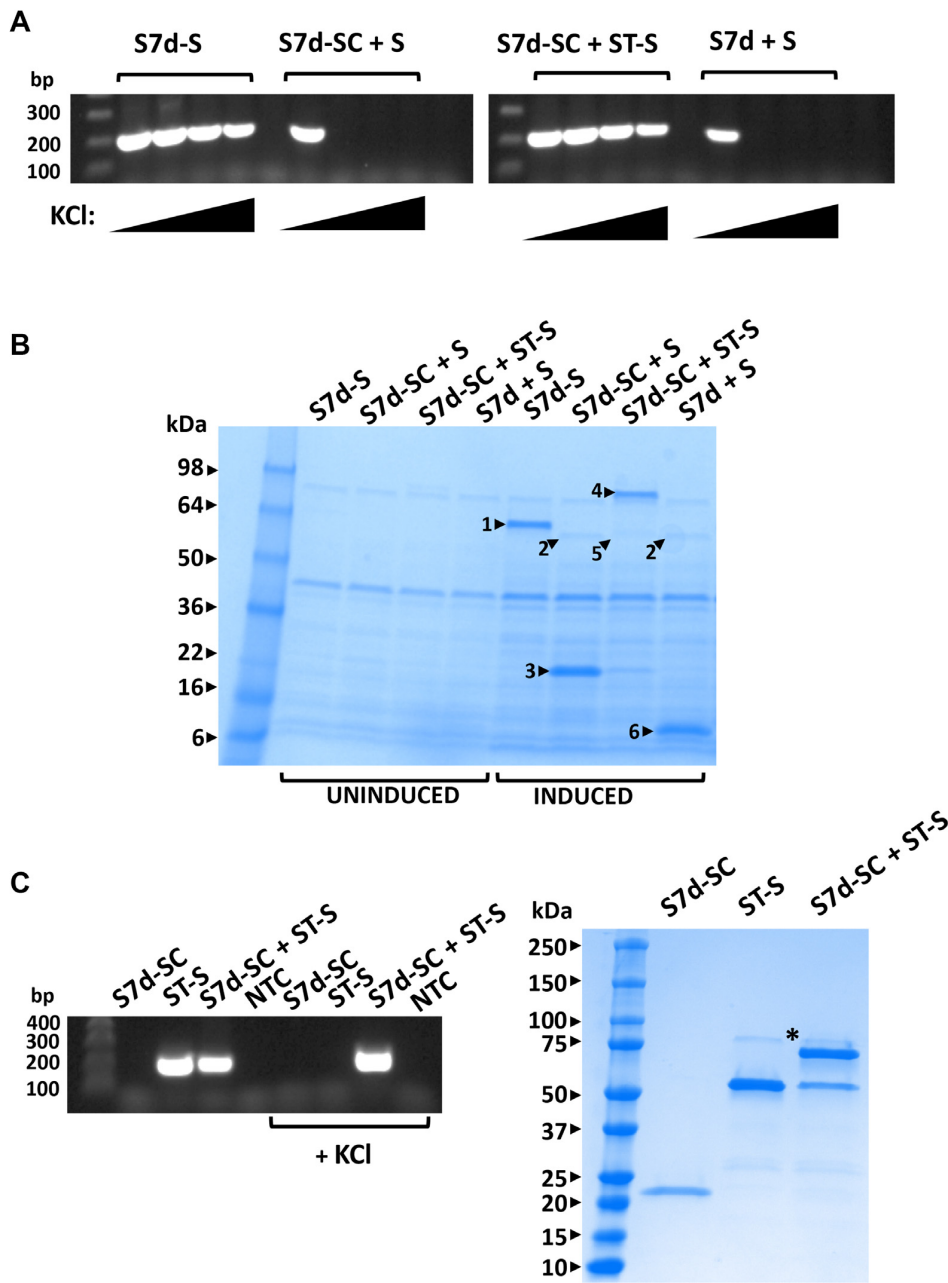
Coupling of Sso7d and Stoffel fragment mediated by SpyCatcher-SpyTag interaction facilitates PCR in high-salt buffer conditions. (**A**) Indicated proteins were (co)-expressed in *Escherichia coli* and cells directly used in PCR reactions with increasing KCl concentrations (0, 100, 200, 300 mM). S7d-S: Sso7d-Stoffel fusion; S7d-SC: Sso7d-SpyCatcher fusion; ST-S: SpyTag-Stoffel fusion; S: Stoffel; *n*=1. Replicate data shown in Figure 1C for S7d-S lanes and 3B, 7B for Sd7-SC + ST-S and S7d-SC + S lanes. (**B**) SDS-PAGE analysis of uninduced/induced *E. coli* cell lysates (co)-expressing indicated proteins. Highlighted bands indicate 1: Sso7d-Stoffel fusion (S7d-S); 2: Stoffel fragment (S); 3: Sso7d-SpyCatcher fusion (S7d-SC); 4: Sso7d-SpyCatcher fusion conjugated to SpyTag-Stoffel fusion (ST-S); 5: SpyTag-Stoffel fusion (ST-S); 6: Sso7d (S7d); *n*=1. Replicate data for S7d-SC + S and S7d-SC + ST-S lanes shown in Figure 7A. (**C**) Indicated proteins (recombinantly expressed and purified) were (co)-incubated for 30 min and an aliquot used in PCR with both normal and high-salt buffer (+ 100 mM KCl). The same reaction mixes were analyzed by SDS-PAGE (right). Highlighted band (*) indicates S7D-SC-ST-S fusion protein; *n*=1.

**Figure 3.**
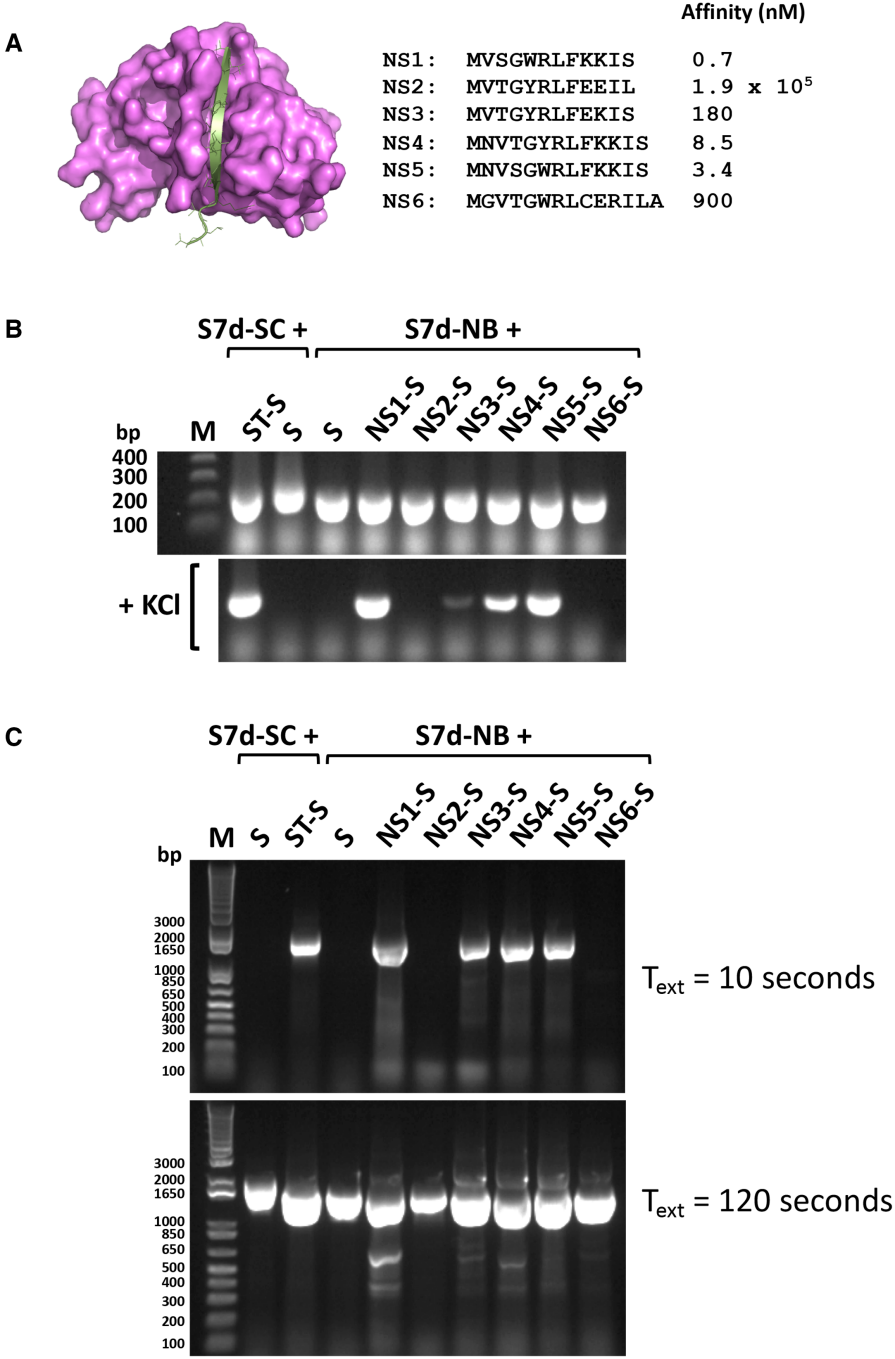
Coupling of Sso7d and Stoffel fragment mediated by reconstitution of split Nanoluc luciferase facilitates PCR in high-salt buffer conditions and accelerated cycling conditions. (**A**) Structure of NanoLuc highlighting the large (magenta) and small (silver) fragments of split Nanoluc (adapted from 5IBO). Peptide sequences of the endogenous (NS6) and engineered small fragments (NS1-NS5) along with affinity constants (8) indicated to the right. (**B**) PCR amplification in absence (top panel) and presence (lower panel) of 100 mM KCl by indicated co-expressed proteins. S7d-SC: Sso7d-SpyCatcher fusion; ST-S: SpyTag-Stoffel fusion; S: Stoffel. S7d-NB: Sso7d-NanoLuc large fragment fusion; NS(1–6)-S: NanoLuc small fragment-Stoffel fusion; *n*= 2 (replicate data shown in Supplementary Figure S1). (**C**) PCR amplification of a 1545 bp fragment by indicated (co) expressed proteins using accelerated (15 s annealing, 10 s extension, upper panel) and normal (30 s annealing, 120 s extension, lower panel) cycling parameters; *n* = 1.

The SpyCatcher-SpyTag protein–peptide pair associate with relatively high affinity to form a complex with exceptional stability due to interlinking isopeptide bond formation (7). Sso7d-SpyCatcher and SpyTag-Stoffel fusion proteins were co-expressed in *E. coli* and polymerase activity assayed by adding cells directly to other standard PCR components and carrying out thermal cycling in buffer with increasing salt concentrations. Covalent association between SpyCatcher and bound SpyTag peptide resulted in an Sso7d-SpyCatcher-SpyTag-Stoffel fusion protein competent for PCR in high-salt buffer (Figure 2A). Control reactions omitting either one or both of the SpyCatcher/SpyTag components did not show any DNA amplification. SDS-PAGE analysis of cell lysates used in PCR confirmed formation of the thermostable Sso7d-SpyCatcher-SpyTag-Stoffel fusion protein (Figure 2B). Similar results were obtained using purified protein components (Figure 2C). Only the reaction comprising Sso7d-SpyCatcher and SpyTag-Stoffel proteins yielded PCR amplicons in high-salt buffer, with formation of the Sso7d-SpyCatcher-SpyTag-Stoffel fusion protein again confirmed by SDS-PAGE. We next replaced the SpyCatcher and SpyTag components with the noncovalently interacting large and small peptide fragments of split NanoLuc luciferase (NB and NS, respectively) (8). A series of small peptide fragments with wide ranging affinities for the large fragment (*K*ds 0.7–1.9 × 10^5^ nM) were fused to Stoffel and individually co-expressed with the Sso7d-large fragment chimera. PCR analysis directly using expressor cells showed a positive high-salt buffer read-out for peptide variants with affinities $ \le$ 180 nM for the large NanoLuc fragment (Figure 3A,B and Supplementary Figure S1). Furthermore, amplicon yields correlated with the reported affinities of the small fragment peptides (8), with maximal polymerase activity observed for the highest affinity peptide (NS1, *K*d = 0.7 nM). We additionally assayed activity of the same panel of expressor cells for amplification of a larger 1545 bp fragment using normal buffer conditions but reduced annealing and extension times (15 and 10 s, respectively) during thermal cycling. Under these conditions, the same subset of salt-tolerant expressor cells yielded the correct amplicon (Figure 3C). Notably, all expressor cells yielded the larger amplicon when longer annealing and extension times (30 and 120 s) were used. Similar results were obtained using recombinant Sso7d-SpyCatcher and SpyTag-Stoffel proteins. Generation of the larger amplicon using a shorter extension time (15 s) only occurred when both proteins were present whilst SpyTag-Stoffel alone was able to yield amplicon using longer (120 s) extension time (Supplementary Figure S2). Therefore, high-affinity interactions can also be assessed using fast cycling conditions that require processivity gains attendant on Sso7d co-localization with polymerase to generate signal.

### Model selections for interacting proteins and peptides using the compartmentalized self-replication (CSR) platform

**Figure 4.**
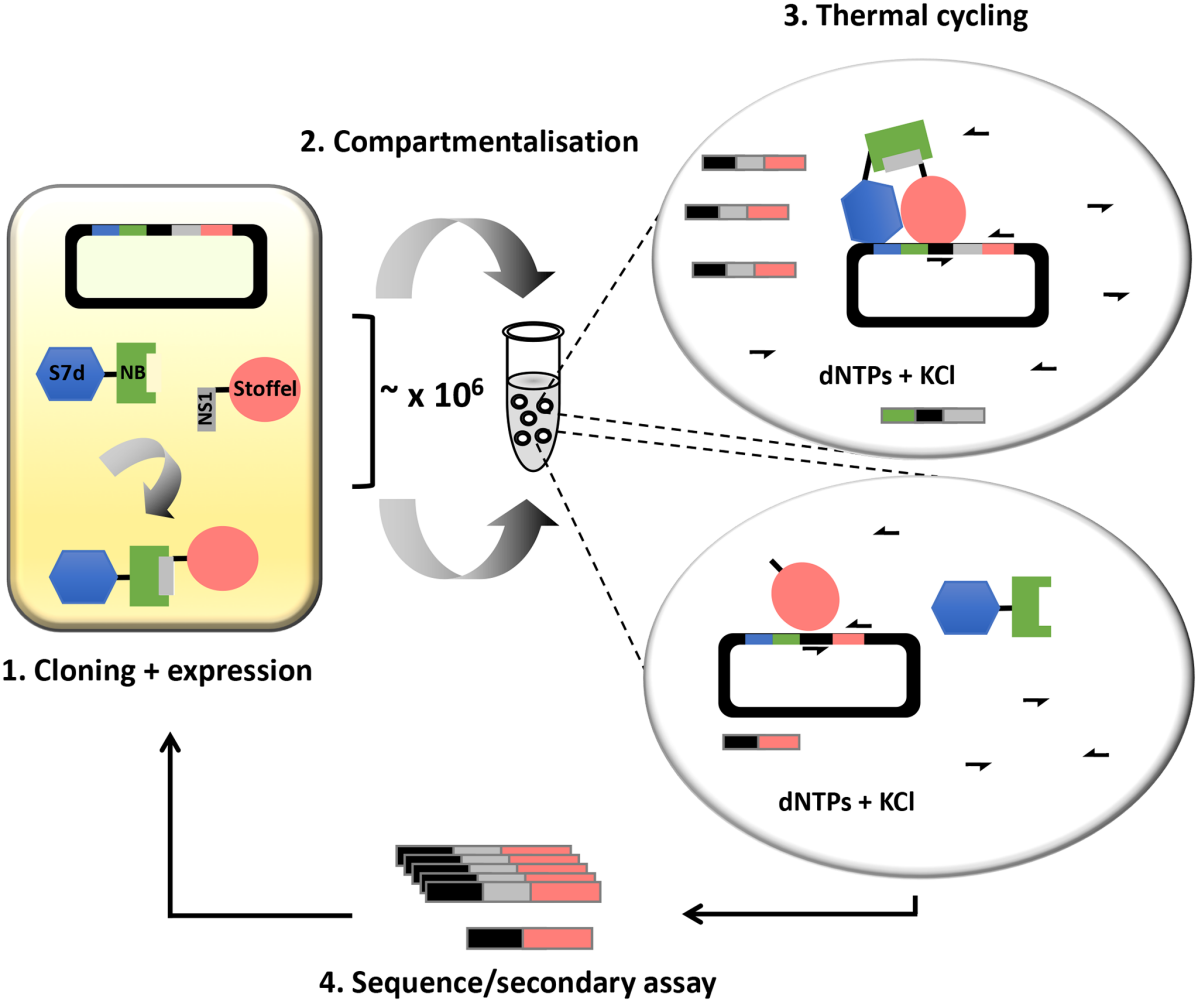
C2HR selection paradigm. (1) Genes encoding a protein (NB) and interacting peptide (NS1) are co-expressed in *Escherichia coli* from a single plasmid as respective fusions to Sso7d (S7d) and Stoffel fragment of Taq polymerase. (2) Cells are clonally segregated into discrete aqueous compartments comprising PCR reagents and high KCl buffer. (3) Thermal cycling lyses cells and gene amplification mediated by specific primers (arrows) is only efficient in compartments hosting an interacting protein–peptide pair (top bubble). Deletion of the peptide gene from the expression plasmid (lower bubble) results in poor amplification due to none co-localization of the Sso7d and Stoffel components. Gene amplification is correspondingly poor in cells co-expressing weak/none interacting protein–peptide pairs when libraries are interrogated. (4) Amplicons are harvested for analysis and/or further rounds of selection.

**Figure 5.**
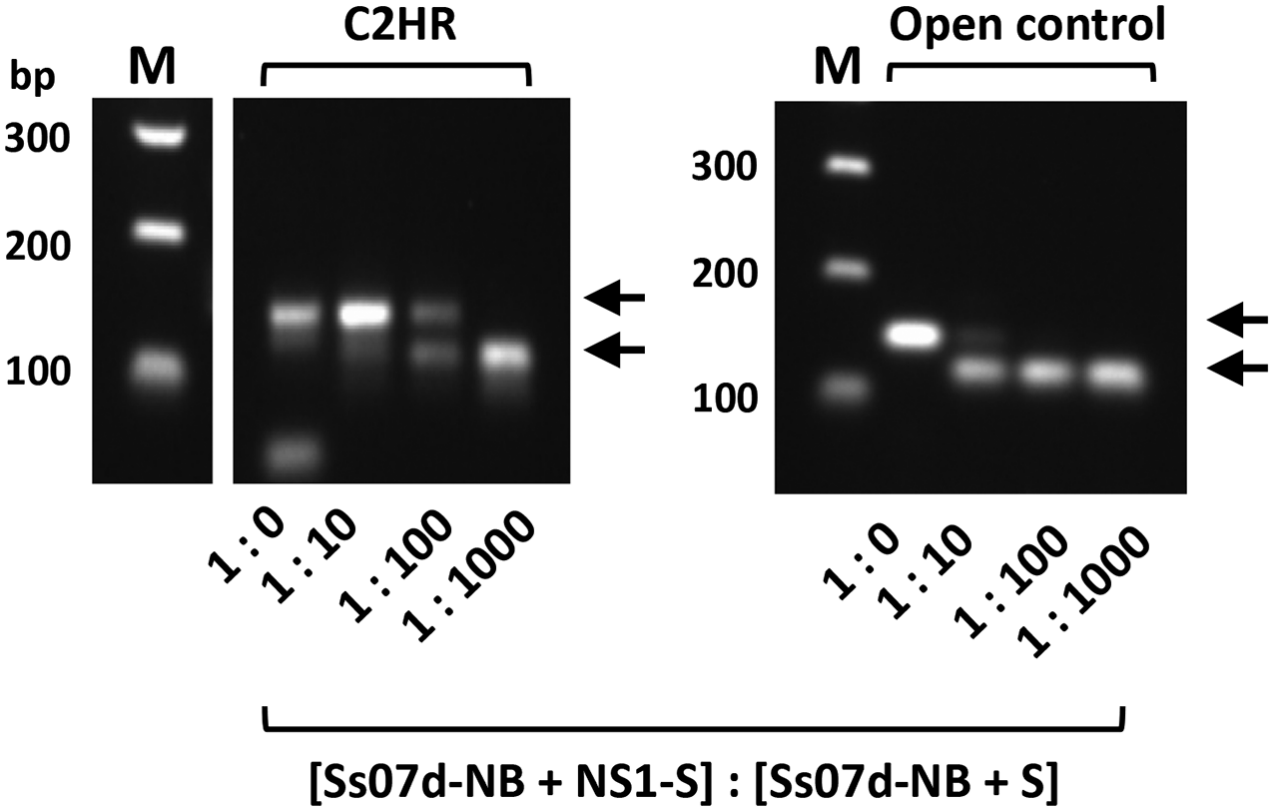
C2HR model selection. *Escherichia coli* cells co-expressing either Sso7d-NB + NS1-S or Sso7d-NB + S were mixed at different ratios prior to emulsification and CSR in high KCl buffer (left panel) or direct PCR in high-salt buffer (open control). Upper arrow indicates amplicon derived from cells expressing Sso7d-NB + NS1-S. Lower arrow indicates amplicon derived from cells expressing Sso7d-NB + S. These bands correspond to the large and small amplicons depicted in Figure 4; *n*=1.

The dynamic read-out of the reporter polymerase was next evaluated in the CSR platform. A test selection was carried out using *E. coli* cells co-expressing either Sso7d-NB + Stoffel or Sso7d-NB + NS1-Stoffel (Figure 4). Cells were mixed at different ratios prior to emulsification and thermocycling in high-salt buffer using a primer pair common to both expression constructs flanking the NS1 cassette. In the absence of emulsification, the Sso7d-NB-NS1-Stoffel complex amplified from both expression plasmid templates as expected (Figure 5). In contrast, C2HR enabled clonal amplification/enrichment of the NS1 cassette in plasmids expressing NS1-Stoffel (upper arrowed band) over those expressing Stoffel only (lower arrowed band). This is readily apparent at the 1:100 ratio of cells, with selection for the NS1 gene cassette occurring only when C2HR is used. The panel of cells co-expressing Sso7d-NB and NS-Stoffel variants (Figure 3) were next combined equally and one round of C2HR carried out. Analysis of only 10 selectants indicated preferential enrichment for the high-affinity NS1 variant (*K*d = 0.7 nM, 5/10 selectants) followed by the next highest affinity variant, NS5 (*K*d = 3.4 nM, 3/10 selectants). The other two selectants encoded the lower affinity NS2 variant. Together, these experiments confirm that C2HR can select for high-affinity interacting protein pairs.

### Selection for functional SpyTag peptide variants using compartmentalized two-hybrid replication (C2HR)

**Figure 6.**
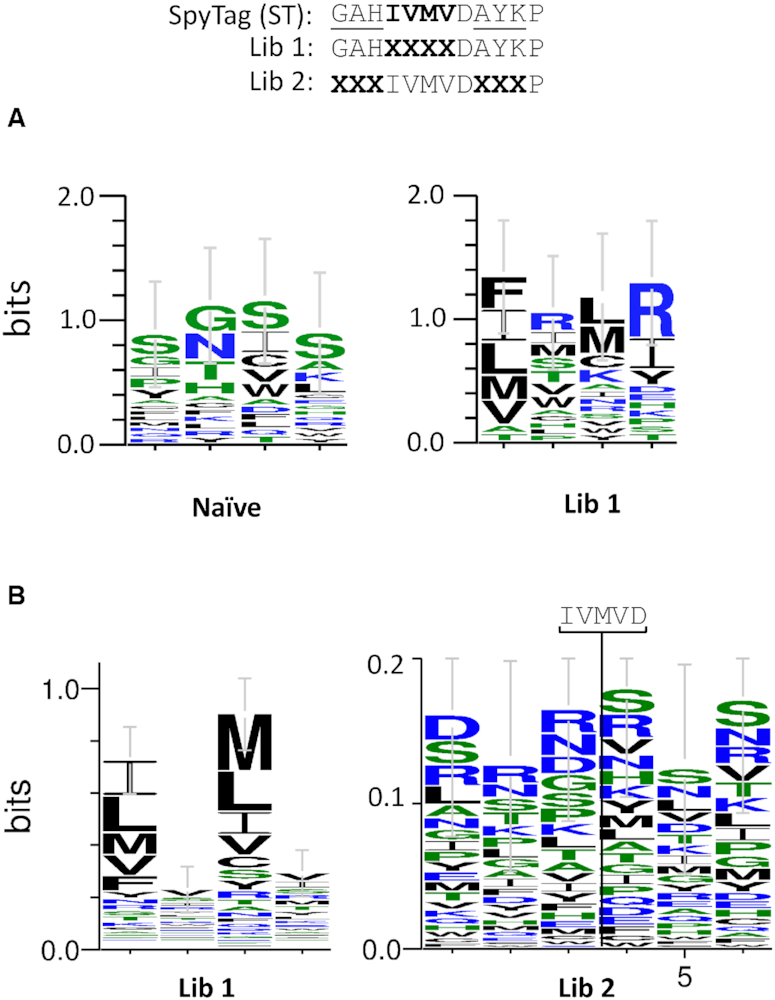
C2HR selection of functional SpyTag and related variants. (**A**) Consensus sequence logos (27) derived from naïve (*n* = 20) and library 1 selectants (top 20 enriched). (**B**) Consensus sequence logos derived from 500 most abundant sequences selected from libraries 1 and 2. Error bars represent ± SD.

We next created a library of SpyTag-Stoffel variants wherein the hydrophobic ‘IVMV’ motif in SpyTag essential for high-affinity interaction with SpyCatcher (Figure 8A) (7) was randomized. This library (Lib 1) was co-expressed in *E. coli* along with Sso7d-SpyCatcher prior to encapsulation in emulsion compartments containing oligonucleotide primers flanking the randomized region of SpyTag along with other requisite PCR components (dNTPs, high-salt buffer). Ten rounds of thermal cycling were carried out to facilitate clonal amplification of genes encoding functional SpyTag core motifs, following which amplicons were harvested and sequenced en masse. This identified selection of 96,400 unique peptide sequences with an average read number of 168. The wild-type ‘IVMV’ motif was the 161st most abundant (16443 reads), indicating positive enrichment (Supporting Data File S2). Consensus motifs (27) highlighting positional frequencies of residues from 20 random sequences from the naïve and the 20 most enriched by selection varied notably, indicating stronger preference for hydrophobic residues in the latter (Figure 6A). Further consensus sequence analysis of the top 500 abundant motifs identified the endogenous ‘IVMV’ motif, and highlighted tolerance for other bulky hydrophobic residues in place of the isoleucine and methionine residues (Figure 6B). These pack into a hydrophobic groove in SpyCatcher and are essential for high-affinity interaction (Figure 8A) (28). Higher sequence variation was tolerated at both valine positions in the motif, again commensurate with structural data showing these residues to project away from the SpyCatcher hydrophobic pocket and contributing less to productive binding interactions.

**Figure 7.**
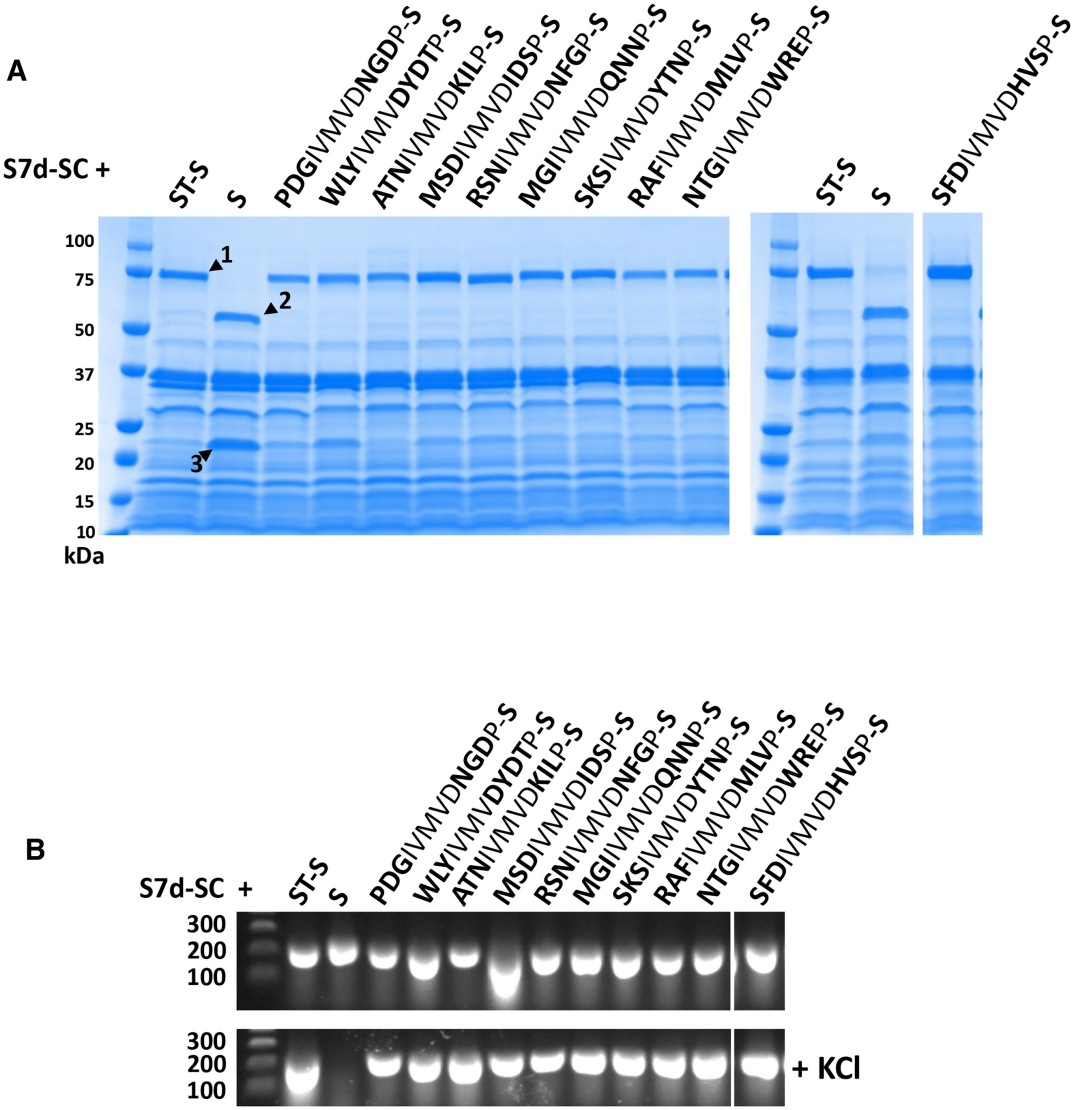
SpyTag variants selected by C2HR retain function as measured by two independent assays. (**A**) Sso7d-SpyCatcher (S7d-SC) fusion protein was co-expressed with Stoffel fragment alone (S) or Stoffel fragment fusions with wild-type SpyTag (ST: GAHIVMVDAYKP) and indicated selectants. Novel residues selected that flank the core ‘IVMVD’ motif of ST are indicated in bold. Highlighted bands represent 1: S7d-SC-ST-S fusion protein; 2: Stoffel fragment; 3: S7d-SC. All selectants yield correct size fusion protein corresponding to wild-type SpyTag control (band 1); *n* = 1. (**B**) The same expressor cells highlighted in (A) were used directly in PCR reactions ± KCl (100 mM). As with wild-type SpyTag, all SpyTag variants enabled PCR in high-salt buffer; *n* = 1.

We next carried out a further single round selection, this time randomizing the three residues either side of the core ‘IVMVD’ motif of SpyTag (Lib 2). The obligate aspartic acid residue in this motif forms the isopeptide bond with lysine 31 in SpyCatcher. Sequencing yielded 160 415 unique peptide sequences with an average read number of 91 (Supporting Data File S2). Endogenous SpyTag with ‘GAH’ and ‘AYK’ flanking motifs was the 52nd most abundant peptide (26 269 reads), again indicating positive selection by C2HR. Notably, no clear consensus motif emerged upon analysis of the top 500 enriched sequences, signifying a higher degree of redundancy for residues flanking the SpyTag ‘IVMVD’ core motif (Figure 6B). This was confirmed by analysis of the top 10 enriched flanking motifs for SpyCatcher binding. All showed a positive, covalent interaction with SpyCatcher as judged by high-salt PCR and SDS-PAGE analysis (Figure 7). We further synthesized biotinylated peptides encoding SpyTag and the top Lib 2 selected variant (STL2: SFDIVMVDHVS) and assayed pull-down of a recombinant target protein (Sso7d-SpyCatcher). As before, the variant showed comparable activity to SpyTag, pulling down a similar amount of the SpyCatcher fusion protein (Supplementary Figure S3). In both the L1 and L2 selections, wild-type SpyTag was present among the top 0.5% most abundant peptide sequences selected after one round, giving an indication of the cut-off threshold for future selections.

### Co-evolution of an interacting protein–peptide pair using C2HR

**Figure 8.**
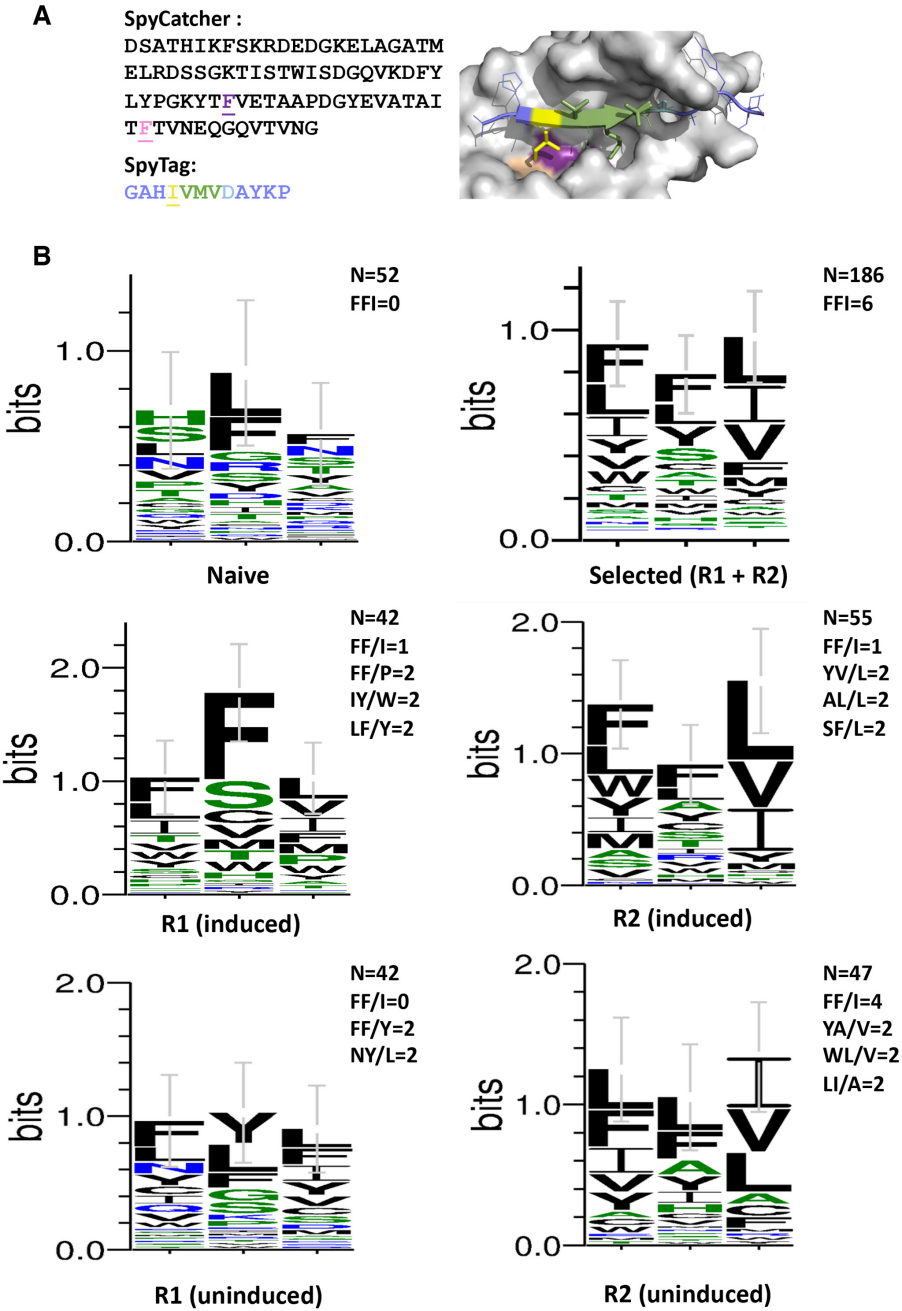
Directed co-evolution of SpyCatcher and SpyTag. (**A**) The two underlined phenyalanine residues in SpyCatcher and the underlined isoleucine in SpyTag were randomized prior to selection. The corresponding positions of these residues (purple, pink and yellow respectively) in the binary complex is shown on the right (adapted from 4MLI) (28). (**B**) Consensus sequence logos for naïve and library selectants after one or two rounds of C2HR. Frequency of endogenous (FF/I) and other enriched motifs indicated. Top right logo denotes aggregate consensus for all round 1 and round 2 sequences. *n* = 52 (naïve), 42 (R1 induced, R1 uninduced), 55 (R2 induced), 47 (R2 uninduced) and 186 (all R1 and R2 selectants). Error bars represent ± SD.

We next investigated co-evolution of both peptide and an interacting partner using C2HR. The isoleucine residue in the core ‘IVMV’ motif of SpyTag packs into a discrete hydrophobic pocket lined by phenylalanines 75 and 92 of SpyCatcher (Figure 8A). These three residues were simultaneously randomized to cover all amino acid combinations and C2HR selection carried out. In contrast to previous selections, the primer pair was chosen to generate amplicons co-encoding interacting SpyCatcher and SpyTag variants during the emulsion PCR phase. We additionally carried out selections using uninduced cells, relying on T7 promoter leakiness to reduce protein levels and potentially increase selection pressure.

After one round of selection using induced cells, 1 out of the 42 selectants analyzed comprised the endogenous FF/I residues at the randomized SpyCatcher/SpyTag positions. Other combinations that were enriched included IY/W, LF/Y and FF/P (2 out of 42 selectants for each). Consensus sequence analysis of all 42 selectants further highlighted preference for hydrophobic residues at the three randomized positions (Figure 8B). In particular, clear selection for the endogenous phenylalanine residues in SpyCatcher was observed. No clear consensus emerged from analysis of 52 random sequences from the unselected library, although there was some inherent bias for phenylalanine and leucine at codon 92 of SpyCatcher. A second round of selection did not lead to enrichment of any specific motif, but clearly enriched for bulky hydrophobic residues at the randomized positions. In the absence of induction, the FFI motif was not observed in any of the selectants analyzed in the first round. It was, however, enriched after the second round (4/47 selectants). As with induced C2HR conditions, clear selection for bulky hydrophobic residues was also observed. The aggregate consensus for all sequences enriched during both rounds (Figure 8B, top right) further emphasizes this preference.

## DISCUSSION

We have described facile detection of protein–peptide interactions through coupling to enzymatic activity of a thermostable nucleic acid polymerase. Whilst we have exemplified using the Stoffel fragment of Taq polymerase, evolutionary conservation of protein–nucleic acid interaction mechanisms (29,30) suggests that other families and classes of polymerase (e.g., DNA/RNA dependent RNA polymerase) could potentially be configured to work in C2HR. As shown, *E. coli* cells co-expressing a protein–peptide pair can be added directly into a PCR tube and interaction validated by assessing amplicon yield after thermal cycling. While end-point PCR was used to validate interactions, more quantitative readouts could be obtained using real-time PCR. Emulsion PCR and other single molecule detection methodologies (31–33) could possibly also be adapted for absolute (i.e., digital) quantification of interacting pairs.

We have further transposed the interaction assay into the CSR directed evolution platform to select for peptide binders using two model interacting peptide–protein pairs. The highest affinity variant of a peptide fragment of split NanoLuc luciferase was readily enriched from a test pool of described peptides with wide-ranging affinities. As next exemplified using the interacting SpyCatcher-SpyTag pair, a much larger repertoire of candidate peptides was interrogated through a single round of C2HR and deep sequencing to rapidly identify binders with a hydrophobic consensus peptide motif comprising the endogenous SpyTag core sequence. Given the irreversibility of the SpyCatcher–SpyTag interaction, it is likely that selection for improved SpyTag variants will require additional selection pressure. This could be introduced by further reducing substrate levels through tighter control of intracellular expression and/or co-expression of competing substrates. Another option is to express SpyTag variants fused to the Stoffel fragment intracellularly, and titrate levels of recombinant Sso7d–SpyCatcher adding during C2HR emulsification of cells. We have also shown directed co-evolution by selection of interacting protein–peptide pairs from a focused co-randomized library. Here, we varied the key isoleucine in SpyTag along with the two phenylalanines in the SpyCatcher hydropbobic cleft that it packs against. Selection yielded the endogenous SpyCatcher and SpyTag residues, and highlighted functional degeneracy, with many other combinations of hydrophobic residues being tolerated. This plasticity has previously been exploited to yield orthogonal SpyCatcher–SpyTag pairs through mutagenesis of the same residue set by conventional screening (34). Further C2HR selections incorporating competitor substrates and deep sequencing could therefore yield many more orthogonal protein–peptide pairs with wide-ranging applications (34–36). Additionally, use of faster cycling conditions to score for productive interactions (Figure 3C and Supplementary Figure S2) would obviate the use of higher salt concentrations during selections, and could be employed to select for protein–peptide interactions under more physiologically relevant conditions. Of pertinent interest would be the study and co-evolution of interactant pairs in clinically relevant virus–host systems (37,38).

The protein–peptide pairs used in this study are inherently thermostable, a pre-requisite for polymerase read-out of interactions by thermal cycling. The SpyCatcher-SpyTag pair has a reported *T*_m_ of 85.4°C, while the large fragment of Nanoluc has a *T*_m_ of 54°C (8,39). The thermal stability of both is likely further elevated through binding to high-affinity peptides and by fusion to the highly thermostable Sso7d so as to enable read-out using thermal cycling at consistently elevated temperatures. This thermostability requirement could be exploited to select for thermostabilizing mutations in peptide-binding proteins (e.g., ScFvs, single domain antibodies), using modulation of cycling parameters (denaturation time, anneal and extension temperatures) to control stringency. C2HR could further be adapted to selections using mesophilic proteins by switching to heat-independent cell lysis protocols such as freeze–thaw and/or enzymatic lysis (40,41). Additionally, the reporter polymerase co-localization paradigm may also be applicable to other DNA transacting enzymes used in amplification protocols such as the phi29 and Bst LF polymerases, both of which have been used in isothermal CSR (40–44). These lower temperature adaptations will also likely be required for detection of weaker, and possibly more physiologically relevant interactions using C2HR.

The C2HR platform can be further adapted to select for other classes of proteins whose activity (in)directly facilitates co-localization of polymerase and processivity factor components. These include peptide ligases belonging to the hydrolase and transglutaminase families and intein domains that regulate protein splicing (45–49). Examples include the transpeptidase Sortase A, capable of ligating short cognate peptide motifs that could be appended to the respective Sso7d and Stoffel fragments. Intein domains can be split into two components, enabling trans-splicing of genetically fused partners (48). Use of Sso7d and Stoffel as partners could therefore facilitate C2HR read-out attendant on reconstitution of intein function.

Nucleic acid modifying enzymes, particularly DNA recombinases could also be engineered by C2HR. In this case, enzyme activity fuses the otherwise split processivity and polymerase gene cassettes, leading to expression of the requisite fusion protein. While this approach has been previously described using other reporter genes (50,51), we anticipate that dynamic read-out afforded by polymerase function will expedite selections.

## Supplementary Material

gkaa933_Supplemental_Files
