# Supplementary material for: Directed co-evolution of interacting protein–peptide pairs by compartmentalized two-hybrid replication (C2HR)
Source: Nucleic Acids Res. 2020 Oct 26;48(22):e128. doi: 10.1093/nar/gkaa933 (PMC7736784; doi:10.1093/nar/gkaa933)
Supplement: gkaa933_Supplemental_Files [file gkaa933_supplemental_files.zip › NAR-Supporting data 1.docx]

**Supporting data 1.**

Amino acid sequences of proteins expressed. Linker residues in fusion proteins are underlined.

**1) Stoffel fragment (S)**

MSPKALEEAPWPPPEGAFVGFVLSRKEPMWADLLALAAARGGRVHRAPEPYKALRDLKEARGLLAKDLSVLALREGLGLPPGDDPMLLAYLLDPSNTTPEGVARRYGGEWTEEAGERAALSERLFANLWGRLEGEERLLWLYREVERPLSAVLAHMEATGVRLDVAYLRALSLEVAEEIARLEAEVFRLAGHPFNLNSRDQLERVLFDELGLPAIGKTEKTGKRSTSAAVLEALREAHPIVEKILQYRELTKLKSTYIDPLPDLIHPRTGRLHTRFNQTATATGRLSSSDPNLQNIPVRTPLGQRIRRAFIAEEGWLLVALDYSQIELRVLAHLSGDENLIRVFQEGRDIHTETASWMFGVPREAVDPLMRRAAKTINFGVLYGMSAHRLSQELAIPYEEAQAFIERYFQSFPKVRAWIEKTLEEGRRRGYVETLFGRRRYVPDLEARVKSVREAAERMAFNMPVQGTAADLMKLAMVKLFPRLEEMGARMLLQVHDELVLEAPKERAEAVARLAKEVMEGVYPLAVPLEVEVGIGEDWLSAKE

**2) Topo V HhH-Stoffel (H-S)**

MKSGRQERSEEEWKEWLERKVGEGRARRLIEYFGSAGEVGKLVENAEVSKLLEVPGIGDEAVARLVPGYKTLRDAGLTPAEAERVLKRYGSVSKVQEGATPDELRELGLGDAKIARILGLRSLVNKRLDVDTAYELKRRYGSVSAVRKAPVKELRELGLSDRKIARIKGIPETMLQVRGMSVEKAERLLERFDTWTKVKEAPVSELVRVPGVGLSLVKEIKAQVDPAWKALLDVKGVSPELADRLVEELGSPYRVLTAKKSDLMRVERVGPKLAERIRAAGKRYVEERRSRRERIRRKLRGAYDVGGGMSPKALEEAPWPPPEGAFVGFVLSRKEPMWADLLALAAARGGRVHRAPEPYKALRDLKEARGLLAKDLSVLALREGLGLPPGDDPMLLAYLLDPSNTTPEGVARRYGGEWTEEAGERAALSERLFANLWGRLEGEERLLWLYREVERPLSAVLAHMEATGVRLDVAYLRALSLEVAEEIARLEAEVFRLAGHPFNLNSRDQLERVLFDELGLPAIGKTEKTGKRSTSAAVLEALREAHPIVEKILQYRELTKLKSTYIDPLPDLIHPRTGRLHTRFNQTATATGRLSSSDPNLQNIPVRTPLGQRIRRAFIAEEGWLLVALDYSQIELRVLAHLSGDENLIRVFQEGRDIHTETASWMFGVPREAVDPLMRRAAKTINFGVLYGMSAHRLSQELAIPYEEAQAFIERYFQSFPKVRAWIEKTLEEGRRRGYVETLFGRRRYVPDLEARVKSVREAAERMAFNMPVQGTAADLMKLAMVKLFPRLEEMGARMLLQVHDELVLEAPKERAEAVARLAKEVMEGVYPLAVPLEVEVGIGEDWLSAKE

**3) Sso7d-STOFFEL (S7d-S)**

MATVKFKYKGEEKEVDISKIKKVWRVGKMISFTYDEGGGKTGRGAVSEKDAPKELLQMLEKQKKGSLPETGGGEAPWPPPEGAFVGFVLSRKEPMWADLLALAAARGGRVHRAPEPYKALRDLKEARGLLAKDLSVLALREGLGLPPGDDPMLLAYLLDPSNTTPEGVARRYGGEWTEEAGERAALSERLFANLWGRLEGEERLLWLYREVERPLSAVLAHMEATGVRLDVAYLRALSLEVAEEIARLEAEVFRLAGHPFNLNSRDQLERVLFDELGLPAIGKTEKTGKRSTSAAVLEALREAHPIVEKILQYRELTKLKSTYIDPLPDLIHPRTGRLHTRFNQTATATGRLSSSDPNLQNIPVRTPLGQRIRRAFIAEEGWLLVALDYSQIELRVLAHLSGDENLIRVFQEGRDIHTETASWMFGVPREAVDPLMRRAAKTINFGVLYGMSAHRLSQELAIPYEEAQAFIERYFQSFPKVRAWIEKTLEEGRRRGYVETLFGRRRYVPDLEARVKSVREAAERMAFNMPVQGTAADLMKLAMVKLFPRLEEMGARMLLQVHDELVLEAPKERAEAVARLAKEVMEGVYPLAVPLEVEVGIGEDWLSAKE

**4) Sso7d-SpyCatcher (S7D-SC)**

MGSSHHHHHHSQDPATVKFKYKGEEKEVDISKIKKVWRVGKMISFTYDEGGGKTGRGAVSEKDAPKELLQMLEKQKKGSGGGSLPETGGSVDDSATHIKFSKRDEDGKELAGATMELRDSSGKTISTWISDGQVKDFYLYPGKYTFVETAAPDGYEVATAITFTVNEQGQVTVNG

**5) SpyTag-Stoffel (ST-S)**

MGAHIVMVDAYKPTKGSTSSMGGEAPWPPPEGAFVGFVLSRKEPMWADLLALAAARGGRVHRAPEPYKALRDLKEARGLLAKDLSVLALREGLGLPPGDDPMLLAYLLDPSNTTPEGVARRYGGEWTEEAGERAALSERLFANLWGRLEGEERLLWLYREVERPLSAVLAHMEATGVRLDVAYLRALSLEVAEEIARLEAEVFRLAGHPFNLNSRDQLERVLFDELGLPAIGKTEKTGKRSTSAAVLEALREAHPIVEKILQYRELTKLKSTYIDPLPDLIHPRTGRLHTRFNQTATATGRLSSSDPNLQNIPVRTPLGQRIRRAFIAEEGWLLVALDYSQIELRVLAHLSGDENLIRVFQEGRDIHTETASWMFGVPREAVDPLMRRAAKTINFGVLYGMSAHRLSQELAIPYEEAQAFIERYFQSFPKVRAWIEKTLEEGRRRGYVETLFGRRRYVPDLEARVKSVREAAERMAFNMPVQGTAADLMKLAMVKLFPRLEEMGARMLLQVHDELVLEAPKERAEAVARLAKEVMEGVYPLAVPLEVEVGIGEDWLSAKE

**6) Sso7d (S7D)**

MATVKFKYKGEEKEVDISKIKKVWRVGKMISFTYDEGGGKTGRGAVSEKDAPKELLQMLEKQKK

**7) Sso7d-Nanoluc large (S7d-NB)**

MGSSHHHHHHSQDPATVKFKYKGEEKEVDISKIKKVWRVGKMISFTYDEGGGKTGRGAVSEKDAPKELLQMLEKQKKGSGGGSLPETGGSVDMVFTLEDFVGDWEQTAAYNLDQVLEQGGVSSLLQNLAVSVTPIQRIVRSGENALKIDIHVIIPYEGLSADQMAQIEEVFKVVYPVDDHHFKVILPYGTLVIDGVTPNMLNYFGRPYEGIAVFDGKKITVTGTLWNGNKIIDERLITPDGSMLFRVTINS

**8) Nanoluc small 1-Stoffel (NS1-S)**

MVSGWRLFKKISGSTSSMGGEAPWPPPEGAFVGFVLSRKEPMWADLLALAAARGGRVHRAPEPYKALRDLKEARGLLAKDLSVLALREGLGLPPGDDPMLLAYLLDPSNTTPEGVARRYGGEWTEEAGERAALSERLFANLWGRLEGEERLLWLYREVERPLSAVLAHMEATGVRLDVAYLRALSLEVAEEIARLEAEVFRLAGHPFNLNSRDQLERVLFDELGLPAIGKTEKTGKRSTSAAVLEALREAHPIVEKILQYRELTKLKSTYIDPLPDLIHPRTGRLHTRFNQTATATGRLSSSDPNLQNIPVRTPLGQRIRRAFIAEEGWLLVALDYSQIELRVLAHLSGDENLIRVFQEGRDIHTETASWMFGVPREAVDPLMRRAAKTINFGVLYGMSAHRLSQELAIPYEEAQAFIERYFQSFPKVRAWIEKTLEEGRRRGYVETLFGRRRYVPDLEARVKSVREAAERMAFNMPVQGTAADLMKLAMVKLFPRLEEMGARMLLQVHDELVLEAPKERAEAVARLAKEVMEGVYPLAVPLEVEVGIGEDWLSAKE

**9) Nanoluc small 2-Stoffel (NS2-S)**

MVTGYRLFEEILGSTSSMGGEAPWPPPEGAFVGFVLSRKEPMWADLLALAAARGGRVHRAPEPYKALRDLKEARGLLAKDLSVLALREGLGLPPGDDPMLLAYLLDPSNTTPEGVARRYGGEWTEEAGERAALSERLFANLWGRLEGEERLLWLYREVERPLSAVLAHMEATGVRLDVAYLRALSLEVAEEIARLEAEVFRLAGHPFNLNSRDQLERVLFDELGLPAIGKTEKTGKRSTSAAVLEALREAHPIVEKILQYRELTKLKSTYIDPLPDLIHPRTGRLHTRFNQTATATGRLSSSDPNLQNIPVRTPLGQRIRRAFIAEEGWLLVALDYSQIELRVLAHLSGDENLIRVFQEGRDIHTETASWMFGVPREAVDPLMRRAAKTINFGVLYGMSAHRLSQELAIPYEEAQAFIERYFQSFPKVRAWIEKTLEEGRRRGYVETLFGRRRYVPDLEARVKSVREAAERMAFNMPVQGTAADLMKLAMVKLFPRLEEMGARMLLQVHDELVLEAPKERAEAVARLAKEVMEGVYPLAVPLEVEVGIGEDWLSAKE

**10) Nanoluc small 3-Stoffel (NS3-S)**

MVTGYRLFEKISGSTSSMGGEAPWPPPEGAFVGFVLSRKEPMWADLLALAAARGGRVHRAPEPYKALRDLKEARGLLAKDLSVLALREGLGLPPGDDPMLLAYLLDPSNTTPEGVARRYGGEWTEEAGERAALSERLFANLWGRLEGEERLLWLYREVERPLSAVLAHMEATGVRLDVAYLRALSLEVAEEIARLEAEVFRLAGHPFNLNSRDQLERVLFDELGLPAIGKTEKTGKRSTSAAVLEALREAHPIVEKILQYRELTKLKSTYIDPLPDLIHPRTGRLHTRFNQTATATGRLSSSDPNLQNIPVRTPLGQRIRRAFIAEEGWLLVALDYSQIELRVLAHLSGDENLIRVFQEGRDIHTETASWMFGVPREAVDPLMRRAAKTINFGVLYGMSAHRLSQELAIPYEEAQAFIERYFQSFPKVRAWIEKTLEEGRRRGYVETLFGRRRYVPDLEARVKSVREAAERMAFNMPVQGTAADLMKLAMVKLFPRLEEMGARMLLQVHDELVLEAPKERAEAVARLAKEVMEGVYPLAVPLEVEVGIGEDWLSAKE

**11) Nanoluc small 4-Stoffel (NS4-S)**

MNVTGYRLFKKISGSTSSMGGEAPWPPPEGAFVGFVLSRKEPMWADLLALAAARGGRVHRAPEPYKALRDLKEARGLLAKDLSVLALREGLGLPPGDDPMLLAYLLDPSNTTPEGVARRYGGEWTEEAGERAALSERLFANLWGRLEGEERLLWLYREVERPLSAVLAHMEATGVRLDVAYLRALSLEVAEEIARLEAEVFRLAGHPFNLNSRDQLERVLFDELGLPAIGKTEKTGKRSTSAAVLEALREAHPIVEKILQYRELTKLKSTYIDPLPDLIHPRTGRLHTRFNQTATATGRLSSSDPNLQNIPVRTPLGQRIRRAFIAEEGWLLVALDYSQIELRVLAHLSGDENLIRVFQEGRDIHTETASWMFGVPREAVDPLMRRAAKTINFGVLYGMSAHRLSQELAIPYEEAQAFIERYFQSFPKVRAWIEKTLEEGRRRGYVETLFGRRRYVPDLEARVKSVREAAERMAFNMPVQGTAADLMKLAMVKLFPRLEEMGARMLLQVHDELVLEAPKERAEAVARLAKEVMEGVYPLAVPLEVEVGIGEDWLSAKE

**12) Nanoluc small 5-Stoffel (NS5-S)**

MNVSGWRLFKKISGSTSSMGGEAPWPPPEGAFVGFVLSRKEPMWADLLALAAARGGRVHRAPEPYKALRDLKEARGLLAKDLSVLALREGLGLPPGDDPMLLAYLLDPSNTTPEGVARRYGGEWTEEAGERAALSERLFANLWGRLEGEERLLWLYREVERPLSAVLAHMEATGVRLDVAYLRALSLEVAEEIARLEAEVFRLAGHPFNLNSRDQLERVLFDELGLPAIGKTEKTGKRSTSAAVLEALREAHPIVEKILQYRELTKLKSTYIDPLPDLIHPRTGRLHTRFNQTATATGRLSSSDPNLQNIPVRTPLGQRIRRAFIAEEGWLLVALDYSQIELRVLAHLSGDENLIRVFQEGRDIHTETASWMFGVPREAVDPLMRRAAKTINFGVLYGMSAHRLSQELAIPYEEAQAFIERYFQSFPKVRAWIEKTLEEGRRRGYVETLFGRRRYVPDLEARVKSVREAAERMAFNMPVQGTAADLMKLAMVKLFPRLEEMGARMLLQVHDELVLEAPKERAEAVARLAKEVMEGVYPLAVPLEVEVGIGEDWLSAKE

**13) Nanoluc small 6-Stoffel (NS6-S)**

MGVTGWRLCERILAGSTSSMGGEAPWPPPEGAFVGFVLSRKEPMWADLLALAAARGGRVHRAPEPYKALRDLKEARGLLAKDLSVLALREGLGLPPGDDPMLLAYLLDPSNTTPEGVARRYGGEWTEEAGERAALSERLFANLWGRLEGEERLLWLYREVERPLSAVLAHMEATGVRLDVAYLRALSLEVAEEIARLEAEVFRLAGHPFNLNSRDQLERVLFDELGLPAIGKTEKTGKRSTSAAVLEALREAHPIVEKILQYRELTKLKSTYIDPLPDLIHPRTGRLHTRFNQTATATGRLSSSDPNLQNIPVRTPLGQRIRRAFIAEEGWLLVALDYSQIELRVLAHLSGDENLIRVFQEGRDIHTETASWMFGVPREAVDPLMRRAAKTINFGVLYGMSAHRLSQELAIPYEEAQAFIERYFQSFPKVRAWIEKTLEEGRRRGYVETLFGRRRYVPDLEARVKSVREAAERMAFNMPVQGTAADLMKLAMVKLFPRLEEMGARMLLQVHDELVLEAPKERAEAVARLAKEVMEGVYPLAVPLEVEVGIGEDWLSAKE
