# Supplementary material for: Directed co-evolution of interacting protein–peptide pairs by compartmentalized two-hybrid replication (C2HR)
Source: Nucleic Acids Res. 2020 Oct 26;48(22):e128. doi: 10.1093/nar/gkaa933 (PMC7736784; doi:10.1093/nar/gkaa933)
Supplement: gkaa933_Supplemental_Files [file gkaa933_supplemental_files.zip › NAR-supporting-figuresREVISION3.pdf]

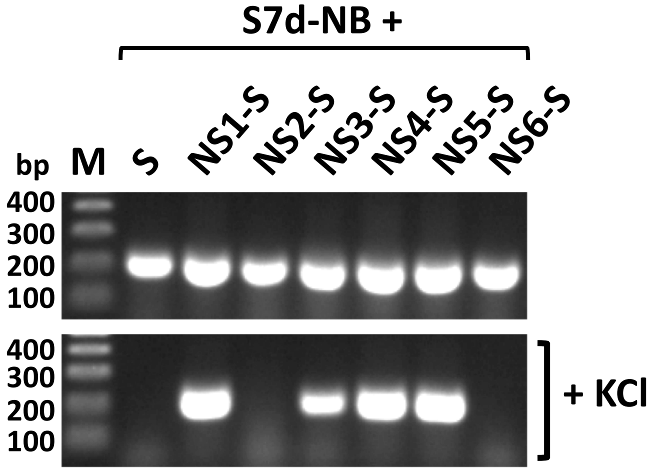

Supporting Figure S1. (A) PCR amplification in absence (top panel) and presence (lower panel) of 100 mM KCl by indicated co-expressed proteins. S: Stoffel. S7d-NB: Sso7d-NanoLuc large fragment fusion; NS(1-6)-S: NanoLuc small fragment-Stoffel fusion.

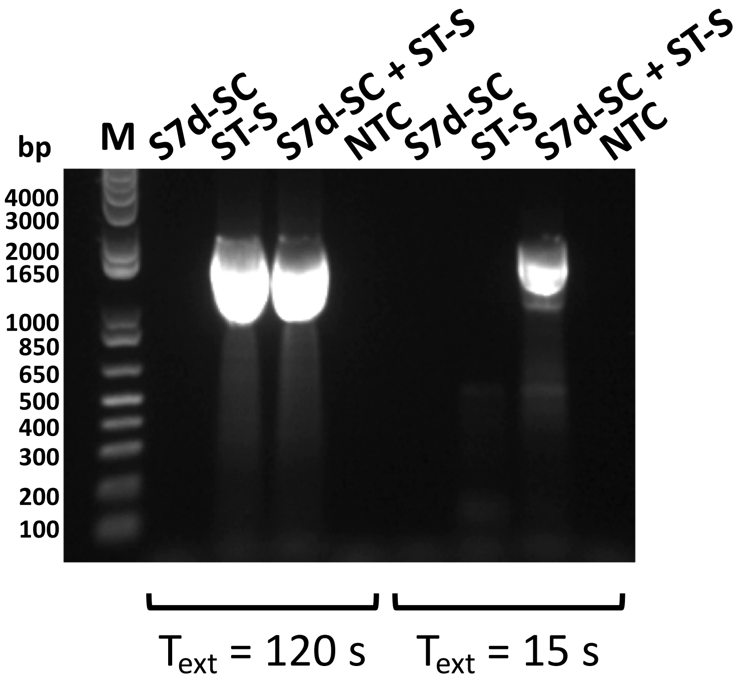

Supporting Figure S2. Indicated proteins (recombinantly expressed and purified) were (co)-incubated for 30 minutes and an aliquot used in PCR amplification of a 1545 bp fragment with either short (15 seconds) or longer (120 seconds) extension time during cycling. n=1.

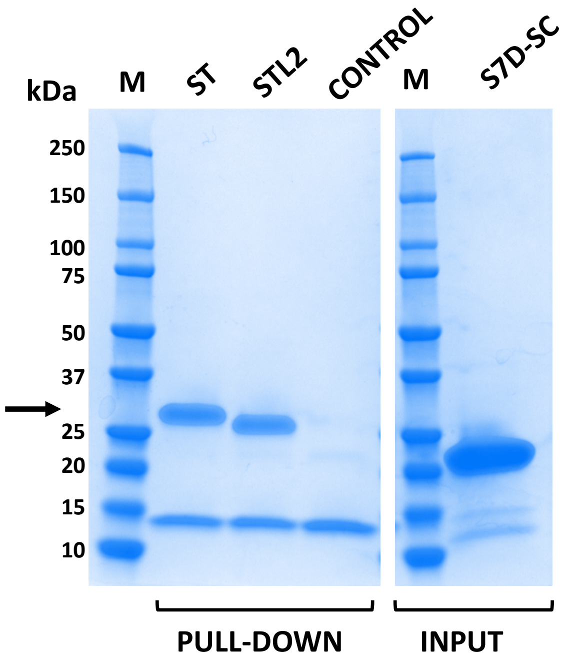

ST : Biotin-SGSGGAHIVMVDAYKPTK

STL2: Biotin-SGSGSFDIVMVDHVSPTK

Supporting Figure S3. Pull-down assay of Sso7D-SpyCatcher protein (S7D-SC) by endogenous (ST) and selected (STL2) biotinylated SpyTag peptides. Covalently bound Sso7D-SpyCatcher protein indicated by arrow in SDS-PAGE gel. Streptavidin beads with no peptides bound used as control. Lower protein band corresponds to streptavidin monomer co-eluted from beads. n=1.
